# Asymptomatic Infant With Atypical SCID and Novel Hypomorphic *RAG* Variant Identified by Newborn Screening: A Diagnostic and Treatment Dilemma

**DOI:** 10.3389/fimmu.2020.01954

**Published:** 2020-09-29

**Authors:** Maria Chitty-Lopez, Emma Westermann-Clark, Irina Dawson, Boglarka Ujhazi, Krisztian Csomos, Kerry Dobbs, Khuong Le, Yasuhiro Yamazaki, Amir A. Sadighi Akha, Deepak Chellapandian, Ben Oshrine, Luigi D. Notarangelo, Gauri Sunkersett, Jennifer W. Leiding, Jolan E. Walter

**Affiliations:** ^1^Division of Pediatric Allergy/Immunology, University of South Florida at Johns Hopkins All Children's Hospital, St. Petersburg, FL, United States; ^2^Laboratory of Clinical Immunology and Microbiology, NIAID, National Institutes of Health, Bethesda, MD, United States; ^3^Department of Laboratory Medicine and Pathology, Mayo Clinic, Rochester, MN, United States; ^4^Cancer and Blood Disorders Institute, Johns Hopkins All Children's Hospital, St. Petersburg, FL, United States; ^5^Division of Pediatric Allergy and Immunology, Massachusetts General Hospital for Children, Boston, MA, United States

**Keywords:** recombinase activating gene (RAG), newborn screening, SCID, asymptomatic infant, immunodeficiency, HSCT

## Abstract

The T-cell receptor excision circle (TREC) assay detects T-cell lymphopenia (TCL) in newborns and is especially important to identify severe combined immunodeficiency (SCID). A spectrum of SCID variants and non-SCID conditions that present with TCL are being discovered with increasing frequency by newborn screening (NBS). Recombination-activating gene (RAG) deficiency is one the most common causes of classical and atypical SCID and other conditions with immune dysregulation. We present the case of an asymptomatic male with undetectable TRECs on NBS at 1 week of age. The asymptomatic newborn was found to have severe TCL, but normal B cell quantities and lymphocyte proliferation upon mitogen stimulation. Next generation sequencing revealed compound heterozygous hypomorphic *RAG* variants, one of which was novel. The moderately decreased recombinase activity of the *RAG* variants (16 and 40%) resulted in abnormal T and B-cell receptor repertoires, decreased fraction of CD3+ TCRVα7.2^+^ T cells and an immune phenotype consistent with the *RAG* hypomorphic variants. The patient underwent successful treatment with hematopoietic stem cell transplantation (HSCT) at 5 months of age. This case illustrates how after identification of a novel *RAG* variant, *in vitro* studies are important to confirm the pathogenicity of the variant. This confirmation allows the clinician to expedite definitive treatment with HSCT in an asymptomatic phase, mitigating the risk of serious infectious and non-infectious complications.

## Introduction

NBS for SCID was initially developed to identify affected infants, preferably at an asymptomatic stage, facilitating medical intervention before life-threatening complications arise (1–5). The assay is based on the absence or low count of T-cell receptor (TCR) excision circles (TREC), a by-product of TCR V (D) J rearrangement in the thymus during early T-cell development, and is universally available in the United States. Aside from SCID, low TREC detected on NBS also allows for the identification of other conditions associated with primary, secondary, and idiopathic forms of T-cell lymphopenia (TCL). Disorders associated with primary TCL include complete or partial DiGeorge syndrome, CHARGE syndrome, trisomy 21, and ataxia-telangiectasia. Examples of secondary TCL are post-surgical extravasation of fluid and lymphocytes, and gastrointestinal malformations resulting in lymphocyte loss. Finally, idiopathic TCL can occur in as many as 20% of screened cases with low TREC and these infants usually require close follow-up (6–8).

Without early intervention, such as HSCT, enzyme replacement therapy (ERT), or gene therapy (GT), a SCID patient is unlikely to survive beyond the first 2 years of life secondary to infections, failure to thrive, or immune dysregulation (4, 8). The great diversity of TCL-related conditions that can be identified via NBS for SCID in an otherwise asymptomatic child may create a degree of uncertainty regarding management strategies. This particularly applies to conditions where variants in SCID-related genes are discovered, but present with variable immune phenotypes.

Since the initiation of NBS for SCID, this condition is no longer defined by clinical symptoms in the majority of cases. Instead, variants of SCID are classified primarily by immune phenotype. The Primary Immunodeficiency Treatment Consortium (PIDTC) diagnostic algorithm subdivides SCID into three categories—typical SCID, atypical SCID, and Omenn syndrome (OS); patients are classified based on total T-cell enumeration, lymphocyte proliferation, presence of maternal T-cells, characteristic phenotypic features, and gene defects (9). SCID is further characterized by specific genotypes that can result in distinct immune phenotypes based on presence or absence of B and NK cells. X-linked interleukin 2 receptor gamma chain (IL2RG) deficiency, adenosine deaminase (ADA) deficiency, and RAG1/2 deficiencies are common genotypes in typical SCID that can be treated with HSCT. RAG1/2 deficiencies are also the most frequent genotypes to cause atypical SCID variants (4, 5).

There is an emerging category of combined immunodeficiencies (CID) with or without immune dysregulation resulting from hypomorphic variants of SCID genes. Partial RAG defects are an example of this phenomenon (10–13). In contrast with RAG1/2 null variants that typically manifest with a T-B- SCID phenotype, hypomorphic RAG deficiency has a broad spectrum of clinical presentations including OS, atypical SCID, combined immune deficiency with granuloma and/or autoimmunity (CID-G/AI), and TCL (13, 14). Characteristically, a patient with partial RAG deficiency and CID has a low naïve T-cell compartment, but relatively preserved B-cell quantities and immunoglobulin levels that may progress to B-cell lymphopenia and hypogammaglobulinemia with age (13). Patients with CID secondary to hypomorphic *RAG* variants often do not meet PIDTC criteria for SCID or atypical SCID. As the *RAG* genes are highly polymorphic and the clinical spectrum of RAG deficiencies is broad, specific functional assays and T cell receptor/B cell receptor (TCR/BCR) repertoire studies are required to establish the link between a novel *RAG* variant and the patient's immune phenotype.

We present an asymptomatic infant who had undetectable TREC on NBS for SCID and was found to carry compound heterozygous *RAG1* variants, resulting in an abnormal immune phenotype.

## Case Presentation

The patient is a 1-week-old male newborn admitted with concern for SCID due to undetectable TREC on NBS (normal range >20 copies/microliter). The patient was conceived by *in vitro* fertilization and born at 35 weeks of gestation. He had no family history of immunodeficiency.

### Initial Evaluation

Initial laboratory evaluation was notable for a T^−^/B^+^/NK^+^ immune phenotype (Table 1). Due to concern for SCID, pharmacologic prophylaxis against infections was initiated per institutional protocol with acyclovir, fluconazole, and pentamidine, as well as intravenous immune globulins (IVIG). The mother was cytomegalovirus (CMV) seronegative and the patient had no detectable CMV viral load by PCR, hence breastfeeding was permitted. Further infectious evaluation confirmed undetectable serum viral loads of HIV, EBV, and adenovirus by PCR.

**Table 1:** Laboratory assessments.

**Age**	**Pre-HSCT**			**Post-HSCT**			**Age appropriate ranges**	
	**1 week old**	**1–2 months**	**3–4 months**	**12 months old**	**15 months old**	**17 months old**	**0–3 months old**	**12–18 months old**
**LYMPHOCYTE SUBSETS**								
WBC	6,500 (L)	2,430		11,600	15,500 (H)	8,570	7,200–18,000	6,400–12,000
Lymphocyte	1,705 (L)	1,370 (L)	1,221 (L)	1,845 (L)	1,395 (L)	2,064	3,400–7,600	3,600–8,900
CD3+ absolute	261 (L)	202 (L)	203 (L)	858 (L)	529 (L)	1,182	2,500–5,500	2,100–6,200
CD4+ absolute	189 (L)	138 (L)	15 (L)	589 (L)	310 (L)	719	1,600–4,000	1,300–3,400
CD8+ absolute	60 (L)	55 (L)	45 (L)	137 (L)	125 (L)	312 (L)	560–1,700	620–2,000
CD19+ absolute	426	608	476	788	433	281 (L)	300–2,000	720–2,600
CD56+ absolute	967	523	538	207	417	574	170–1,100	180–920
CD4/CD45RA absolute and % of total CD4		82 (L) (60%)	87 (L) (60%)				1,200–3,700	1,000–2,900
CD8/CD45RA absolute and % of total CD8		40 (L) (72%)	37 (L) (82%)				450–1,500	490–1,700
**IMMUNOGLOBULINS**								
Unit: mg/dl								
IgG	1,000	608	570	514	605	569	251–906	345–1,213
IgM	27	27		49	57	120	20–87	43–173
IgA	<7 (< L)	<7 (L)		81	118	181 (H)	1.3–53	14–106
**LYMPHOCYTE PROLIFERATION**								
**EdU (thymidine analog) incorporation**								
Lymphocyte viability, LPM		(L) 64.5					>74.9	
PWM-induced, CD45		33					>4.4	
PWM-induced, CD3		42.1					>3.4	
PWM-induced, CD19		6.4					>3.8	
PHA-induced, CD45		75.1					>49.8	
PHA-induced, CD3		89.6					>58.4	
**3H-thymidine incorporation**								
PHA- induced, CMP			74,853		147,025	223,240	3,700–265,00	73,700–265,000
PHA- induced, SI			536		355	1,432		
Con-A-induced, CMP			61,045		82,251	92,041	6,100–283,00	46,100–283,000
Con-A-induced, SI			437		199	591		
PWM-induced, CMP			44,852		62,143	47,668	9,100–125,00	29,100–125,000
PWM-induced, SI			321		151	307		
**NKC, Pct CYTOTOXICITY**								
NKC, Pct cytotoxicity 50:1		25						
NKC, Pct cytotoxicity 25:1		24						
NKC, Pct cytotoxicity 12:1		15						
NKC, Pct cytotoxicity 6:1		11.5						
NKC, CD16/56 positive		27 (H)						

*WBC, white blood cell count; PWM, pokeweed mitogen; PHA, phytohemagglutinin; Con-A, concanavalin A; NKC, natural killer cells; SI, stimulation index*.

**Figure 1:**
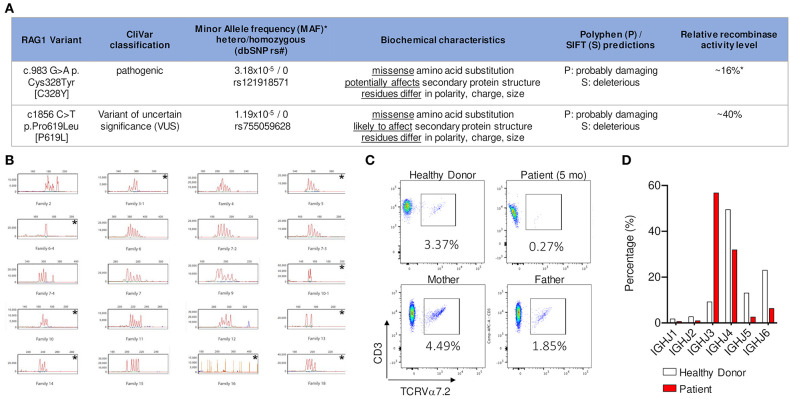
**(A)** NGS identified two heterozygous missense variants in trans in *RAG1*. Relative recombination activity level of the two alleles [16% (ref 16) and 40% (novel variant, measured in this study)] predict an overall relative recombinase activity of 28% that is characteristic of CID-G/AI phenotype (*based on gnomAD 12/2019). **(B)** CD3+ T cells analyzed by spectratyping using 28 probes to measure CDR3 length variability in 23 TCRVβ families and sub-families. Families 2 to 18 are selected for representation. The following families demonstrated an oligoclonal distribution, i.e., <5 peaks: 3–1, 5, 6–4, 10–1, 10, 13, 14, and 18; family 16 had no peak. Each is identified with an * in the upper right corner. **(C)** Detection of Vα7.2 segment in CD3+ T lymphocytes by flow cytometry shows that the patient had near absence of CD3+T cells expressing the TCR Vα 7.2 chain [encoded by a distal V TCR locus] compared to healthy control and family members (measured in collaboration with the NIH). **(D)** Frequency of individual IGHJ gene usage of memory B cell receptor repertoire shows decrease in usage of the distal IGHJ5 and IGHJ6 gene elements in patient compared to age matched healthy donor.

Subsequent evaluation at 1 and 3 months of age revealed that profound T cell lymphopenia (TCL) persisted (<300 cells/μl) and the proportion of naïve CD45RA^+^CD4^+^ T-cells was below normal (60%, normal >90%). Maternal engraftment was excluded by short tandem repeats (STR) analysis. A microarray resulted normal. The patient continued to maintain remarkable CD4^+^ and CD8^+^ lymphopenia; however, B and NK cell quantities remained within normal range (Table 1). After the initial IVIG dose, the patient was transitioned to subcutaneous immunoglobulin replacement therapy (IgRT), which maintained IgG levels within normal range. IgA level was consistently low (<7 mg/dL) and IgM level remained normal during this time (Table 1). Lymphocyte proliferation to the mitogens phytohemagglutinin (PHA) and pokeweed mitogen (PWM) measured at 1 month of age by chemically labeled EdU (thymidine analog) incorporation method were normal. At 3 months of age, lymphocyte proliferation to PHA, concanavalin A (ConA), and PWM measured by radioactive thymidine incorporation method were normal as well (Table 1). TCR Vβ spectratyping was abnormal: of the 28 probes used for 23 TCR Vβ families and sub-families, one had no peak, 8/28 (29%) demonstrated oligoclonality (i.e., <5 peaks), and 15/28 (53%) showed a polyclonal, non-Gaussian distribution (Figure 1B).

Genomic DNA was subjected to target enrichment using hybridization capture with a custom bait pool and sequenced using Illumina sequencing chemistry. A validated bioinformatics pipeline incorporating community standard and custom algorithms was used to simultaneously identify sequence changes and exonic deletions/duplications. Next generation sequencing identified two heterozygous missense variants in trans in *RAG1* (*RAG1* c.983G>A p.Cys328Tyr [C328Y] and *RAG1* c.1856C>T p.Pro619Leu [P619L]). The first variant had been previously published as a homozygous trait in a patient with OS and was also present in the patient's father, who was a heterozygous carrier (15). The second variant was a novel variant of uncertain significance (VUS); the patient's mother was a heterozygous carrier for this variant. These findings confirmed the compound heterozygous status of the patient.

### Evaluation of Rag Variants by Population Database Frequency, *in silico* Analysis, and Recombinase Activity

*RAG1* C328Y has not been linked to any specific ethnicity and has a low mean allele frequency (MAF) in heterozygous form 1/31,392 (3.2e-5) (gnomAD database, June 2020). This non-conservative amino acid substitution could potentially affect secondary protein structure, as these residues differ in polarity, charge, size, and/or other properties. Polyphen and SIFT software programs predict this substitution as probably damaging and deleterious, respectively. *In vitro* relative recombinase activity determined by functional studies has previously shown that C328Y has reduced, but not absent, recombinase activity (16%) (16, 17). Therefore, this variant was established as a likely pathogenic hypomorphic variant.

The pathogenicity of the *RAG1* P619L variant has not previously been published or fully evaluated. Population studies detected this variant in South Asian populations with a MAF of 2/30,782 (6.5e-5) and a European cohort with a MAF of 1/113,661 (8.7e-6), with an overall MAF of 3/251,358 (1.19e-5) (gnomAD database, June 2020) (18). This non-conservative amino acid substitution is likely to affect secondary protein structure, as these residues also differ in polarity, charge, size, and/or other properties. Indeed, Polyphen and SIFT predict this variant as probably damaging and deleterious, respectively. Relative recombinase activity was previously unpublished for this variant and so was evaluated using a previously described platform (17). Relative recombinase activity of the *RAG1* P619L variant was reduced at 40%. In summary, based on the patient's clinical phenotype, repertoire studies, analysis of predicted genotype-phenotype implications, and relative recombinase activity measurement, it is likely that the *RAG1* P619L variant should be reclassified from a VUS to a likely pathogenic hypomorphic variant. Considering one variant had 16% predicted activity and the variant on the other allele 40% predicted activity, the calculated average recombinase activity is 28% (Figure 1A).

### TCR and BCR Repertoire Data

TCR/BCR repertoire studies are an *in vivo* readout for deficiency in V (D) J recombination activity. In particular, reduced use of distal elements can reflect decreased capacity for *RAG* recombinase activity (19). In comparison to healthy controls, there was also a dramatic decline in usage of the distal *IGHJ5* and *IGHJ6* gene elements (BCR), suggesting an abnormal V (D) J recombination consistent with partial *RAG* deficiency (Figure 1D). Detection of Vα7.2 segment (TCR) in CD3^+^ T lymphocytes and/or mucosa-associated invariant T-cells (MAIT) is a rapid method for evaluating decreased usage of distal TCRαV (*TRAV*) gene segments, which indicates a V (D) J recombination defect (20). Indeed, in our case we noted a decrease in CD3^+^ TCRVα7.2^+^ lymphocytes compared to controls (0.27% vs. 1.85–4.49%) (Figure 1C). The TCR/BCR repertoire studies, recombinase activity assay, and specific flow cytometric testing for distal Vα7.2 element, all support a V (D) J recombination defect with partial RAG deficiency as the underlying cause of disease.

### Additional Testing

Lastly, autoantibody testing targeting IFNα, IFNω, and IL-12 was performed by ELISA, as presence of these autoantibodies have been associated with immune dysregulation in partial *RAG* deficiency (12, 21). These anti-cytokine antibodies were not detected in our patient. Increased natural killer (NK) cell cytotoxicity has been associated with increased rate of graft rejection after HSCT in RAG deficient patients (22). In our patient, NK cell cytotoxicity performed at 2 months of age was normal (Table 1).

### Therapeutic Approach

The immune phenotype of our patient included absent TREC, TCL, normal T cell proliferation to mitogens, normal NK cell cytotoxicity, and oligoclonality with reduced relative recombinase activity. Based on his immune abnormalities, HSCT was considered as definitive treatment. The patient lacked an HLA-identical sibling, but several 10/10 HLA-matched unrelated donors (MUD) were available. The patient remained on anti-microbial prophylaxis and protective isolation, infection-free until transplant. At 5 months of age, he underwent HSCT with unmanipulated bone marrow from CMV negative 10/10 MUD (5.56 million CD34+ cells per kg of recipient body weight) with reduced intensity conditioning regimen of sub-myeloablative busulfan, fludarabine, and alemtuzumab, with a busulfan target AUC of 3,500–4,000 μM x min/L (23). Graft versus host disease (GVHD) prophylaxis included tacrolimus with a goal trough level of 10–15 ng/mL and a short-course of methotrexate given on days +1, +3, +6, and +11. The early post-HSCT period was without major complications except for *Enterobacter* bacteremia and *Clostridium difficile* colitis that resolved with antibiotics. The patient had platelet engraftment on day +22 and neutrophil engraftment on day +27 after HSCT. He demonstrated 100% donor chimerism in whole blood, myeloid, and NK lineages on day +27 after transplant and 100% donor T-cell chimerism on day +35.

### Follow-Up

His post-transplant course was complicated by skin-only stage I GvHD that responded to systemic steroids and tacrolimus, and topical immunomodulating agents tacrolimus 0.03% ointment and hydrocortisone 1% cream. At 12 months of age, the patient had an absolute lymphocyte count of 1,895 cells/μL, with 858 T-cells/μL and 788 B-cell cells/μL. Over the past 2 years, he has maintained >90% donor T-cell chimerism, and 30–70% donor B-cell chimerism. The patient was weaned from IgRT 10 months after HSCT (Table 1). He has remained infection-free, off anti-microbial prophylaxis, has developed no signs of autoimmunity or immune dysregulation, and is thriving.

## Discussion

Our case exemplifies the diagnostic and treatment challenges for patients with severe TCL, SCID-related gene defects (i.e., partial *RAG* deficiency), and atypical immune phenotype. The patient's normal B-cell count and immunoglobulin levels except for IgA, higher preserved fraction of naïve CD4+ (60%) and CD8+ (72–82%) T-cells, and normal lymphocyte proliferation with mitogen stimulation and reduced relative recombinase activity of 28% are unusual for most variants of SCID.

While multiple classifications exist for SCID and related disorders that include guidance from PIDTC, European Society for Immunodeficiencies (ESID) and International Union of Immunological Societies (IUIS) (24), these criteria are not applicable to all patients with SCID. Our patient's absolute CD3 T cell count meets the threshold for typical SCID based on PIDTC criteria. However, lymphocyte proliferation is normal, whereas it is very low in typical SCID and leaky SCID/Omenn syndrome according to the PIDTC criteria. He does not meet ESID criteria for typical SCID either, since he was asymptomatic and did not have an affected family member (25). Because of the patient's severe TCL, he was sequestered from birth until HSCT to prevent infections.

Classical SCID with RAG deficiency typically causes a T-B- phenotype as null RAG activity will impede functional TCR/BCR development and thus jeopardizes T and B cell survival. In cases of hypomorphic variants with partially preserved recombinase activity, T and B cells may survive with a restricted TCR/BCR repertoire. *RAG* genes are also highly polymorphic; therefore, all novel variants require confirmation of pathogenicity. This can be done through BCR or TCR repertoire studies and/or *in vitro* recombination assays (17, 26, 27).

Among the repertoire studies available to establish the significance of a *RAG* variant, detection of TCRVα7.2 segment in CD3+ T lymphocytes and/or MAIT is the most rapid and clinically feasible method for evaluating reduced usage of distal *TRAV* segments, indicating a V (D) J recombination defect or impaired thymocyte survival. Notably, TCRVβ spectratyping reflects skewed repertoire based on CDR3 length, commonly seen in SCID variants, but does not reflect use of distal elements, therefore would not inform of V (D) J recombination defects.

Early diagnosis of patients with RAG deficiency is of high importance. If left undiagnosed and untreated, these patients develop severe and often fatal complications of immune dysregulation and/or infections (21, 28, 29). However, there are also reports of the same compound heterozygous *RAG* variants resulting in variable phenotype and disease severity. The cases of two siblings, one with CID and the other with mild T-cell lymphopenia (30), and two adults with the same genetic defect, but highly divergent phenotype (antibody deficiency with mild vs. multiple autoimmune conditions) illustrate this point [patients 12 and 13 from (14) cohort]. Anti-cytokine antibodies are associated with immune deficiency, autoimmunity and/or immune dysregulation, especially in patients with partial RAG deficiency with severe herpesvirus infections (12, 21). Although tested, our patient had not yet developed anti-cytokine antibodies, presumably due to his young age and lack of infectious triggers.

Patients with partial RAG deficiency including atypical SCID and CID phenotype are increasingly recognized in young children and adults after severe infections (13, 14, 21). As with SCID, multiple classification systems for primary CID disorders exist, complicating diagnostic and management decisions. Roifman et al. distinguished CID from SCID based on a total CD3+ T-cell count of >500/μl (31). However, our patient had considerably <500 T cells/μl. In the cohort study of 103 patients by Roifman et al., only 5 had a *RAG-*related condition with OS or SCID phenotypes. All patients had low proliferation to mitogens and low TREC. Our patient also had low TREC but normal mitogens. The conclusions of the report implied that patients with partial RAG deficiency may be detected by NBS, further exemplified in our case. The 2019 ESID criteria for diagnosis of CID requires a symptomatic patient (infections, immune dysregulation) or history of affected family members with immune phenotype of 2 of the 4 parameters (low CD3, CD4 or CD8 T cells; low naïve CD4 and/or CD8 T cells; elevated γδT-cells; or reduced proliferation to mitogen or TCR stimulation) (25). Our patient meets the first two immunophenotypic criteria (note: γδT-cells were not measured) but not the clinical criteria as he was protected from infection by his environment and has no family history of this condition. The ESID 2019 criteria do not discuss underlying genetic defects for CID patients. The IUIS 2020 classification does list genetic defects for CID and does not include *RAG* deficiency (32) in the immune dysregulation group, where the CID-G/AI phenotype would best belong (24). The average recombinase activity of our patient (28%) is consistent with CID but not with typical SCID or OS (<10%) where there is typically lower activity (17, 26). Upon completing extensive immunological testing, our patient appeared to have an atypical SCID phenotype that shares several features with CID.

With the introduction of NBS for SCID, HSCT for asymptomatic SCID infants is increasingly utilized. Our patient had a unconventional immune phenotype with overlap between atypical SCID and CID that influenced the decision and design of the conditioning and transplant process. It is well-established that symptomatic patients with infectious and autoimmune complications, and CID immune phenotype secondary to *RAG* deficiency, do not respond well to immune modulation and ultimately require HSCT (13). However, there are no case studies nor established guidelines available on how to proceed with an asymptomatic infant with extreme TCL but normal T cell proliferation, and mostly normal humoral immunity. The decision to pursue immune reconstitution in hypomorphic *RAG* variants is controversial given the lack of HSCT studies in this particular population of TCL (33). Speckmann et al. published the therapeutic perspective of 51 pediatric patients (median age 9.6 years) with variants of symptomatic atypical SCID and CID characterized by infections and/or immune dysregulation and reduced, but not absent, T-cell immunity (34). The immune phenotype of these patients was not severe enough for an unambiguous early transplant decision. In this study, 29 (56%) of the 51 patients had mild disease, and six were transplanted for “prophylactic reasons” [i.e., family history or because the genetic diagnosis (DCLEREC1, LIGIV, IL2RG, NBN) was considered an HSCT indication].

Because severe T cell lymphopenia predisposes to serious infections, and hypomorphic *RAG* variants are linked to severe autoimmune and inflammatory complications in addition to infections, HSCT was performed electively at 5 months of age for our patient, taking into account our institutional experience and the paucity of data on timing for conditioning in this specific population. Conditioning regimens are increasingly used and streamlined for asymptomatic SCID, leaky SCID infants, and CID patients with *RAG* deficiency in the United States (4, 15, 33, 35, 36). Overall, using chemotherapy-based conditioning confers the advantage of facilitating T and B-cell reconstitution compared to unconditioned transplants in which B-cell dysfunction can persist with engraftment (37). The overactivated T and NK cell compartments should also be considered in the conditioning process and, in this case, inform the decision to use alemtuzumab and pre-transplant immunoablation (38).

Our case underscores the opportunity that SCID-NBS may have in capturing patients with overlapping immune phenotypes before the full spectrum of infectious and non-infectious complications develops. We also demonstrated that the pathogenicity of novel *RAG* variants requires high scrutiny with several methods. Awareness of the multiple presentations of immune deficiency with *RAG* variants is necessary for proper care of patients with this condition. HSCT can be considered for an asymptomatic infant with severe TCL but normal T cell proliferation and B cell number; however, further studies are needed to improve the knowledge of the natural course of the disease.

## Ethics Statement

Written informed consent was obtained from the minor's legal guardian for the publication of any potentially identifiable images or data included in this article.

## Author Contributions

MC-L, EW-C, and ID conceived the presented idea. BU, KL, KD, KC, YY, and AASA performed functional assays and assisted with data interpretation. DC and BO verified the analytical methods used and reviewed the clinical information presented. LN, GS, JL, and JW encouraged to describe this patient's entity and clinical course in the context of internationally accepted guidelines and supervised the findings of this work. All authors discussed the results and contributed and agreed to the final manuscript. All authors contributed to the article and approved the submitted version.

## Conflict of Interest

The authors declare that the research was conducted in the absence of any commercial or financial relationships that could be construed as a potential conflict of interest. The handling editor declared a past co-authorship with one of the authors with several of the authors JL and LN.
